# Biological corneal inlay for presbyopia derived from small incision lenticule extraction (SMILE)

**DOI:** 10.1038/s41598-018-20267-7

**Published:** 2018-01-30

**Authors:** Yu-Chi Liu, Ericia Pei Wen Teo, Heng Pei Ang, Xin Yi Seah, Nyein Chan Lwin, Gary Hin Fai Yam, Jodhbir S. Mehta

**Affiliations:** 1 Tissue Engineering and Stem Cell Group, Singapore Eye Research Institute, Singapore, Singapore; 2 Singapore National Eye Centre, Singapore, Singapore; 3 Duke-NUS Graduate Medical School, Singapore, Singapore; 4 School of Material Science & Engineering and School of Mechanical and Aerospace Engineering, Nanyang Technological University, Singapore, Singapore

## Abstract

Corneal inlays are a relatively new treatment option for presbyopia. Using biological inlays, derived from lenticules extracted from small incision lenticule extraction, may offer advantages over commercialized synthetic inlays in the aspect of biocompatibility. We conducted a non-human primate study to evaluate the safety, predictability, efficacy and tissue response after autogeneic, decellularized xenogeneic and xenogeneic lenticule implantation. The lenticule implantation effectively resulted in central corneal steepening (simulated keratometric values increased by 1.8–2.3 diopters), central hyper-prolate changes (asphericity Q values changed by −0.26 to −0.36), corneal anterior surface elevation (7.7–9.3 μm) and reasonable effective zone (1.5–1.8 times of the lenticule physical diameter), with no differences among the three groups. Slit lamp microscopy, transmission electron microscopy, confocal microscopy, histology and immunohistochemistry analyses confirmed the biocompatibility of the autogeneic and decellularized lenticules, whereas one eye in the xenogeneic group developed corneal stromal rejection during the study period. Our results showed that lenticule implantation has the potential for the management of presbyopia, and provide the basis for future clinical studies. The decellularization process may increase the potential utilization of lenticules without changing the efficacy.

## Introduction

Presbyopia affects individuals older than 40 years and is the most common refractive error^1^. It currently affects approximately 2 billion people worldwide, and it is expected that 2.1 billion people will be affected worldwide by 2020^2^. Presbyopia is a significant burden on productivity, and correction of presbyopia would have a significant impact on productivity^3^. Surgical managements for presbyopia correction include monovision, multifocal intraocular lenses, conductive keratoplasty and corneal presbyopic laser surgery^4,5^. However, no single technique has been accepted as a standard for the treatment of presbyopia.

Corneal inlays are a relatively new treatment option for presbyopia. Currently, there are four commercially available corneal inlays^4^, and these inlays have different principles of mode of action. Among them, the Raindrop inlay (ReVision Optics, Lake Forest, CA, USA) is designed to reshape the central anterior corneal surface, creating a hyper-prolate region of increased power for focusing on near and intermediate objects^4^. Clinical studies have shown that the Raindrop inlay provides significant improvement in patients’ near and intermediate visual performance, with no significant change in binocular distance vision or contrast sensitivity^6^. However, all current available inlays are made of synthetic materials and may be associated with complications pertinent to stromal inflammatory response, such as corneal haze^6^, or changes in the metabolic environment and ion transport in the stromal space that lead to anterior stromal thinning or keratolysis^7,8^. It was reported that central corneal haze was observed in 14% of patients who underwent the Raindrop implantation, although the majority of haze could resolve with the treatment of topical steroids^6^. The use of biological inlays could be a method to address the problems related to the tissue reaction resulting from the insertion of synthetic inlays.

Small incision lenticule extraction (SMILE) is a small-incision, femtosecond laser refractive lenticule extraction (ReLEx) procedure. It has become clinically available in Europe and Asia as an alternative to laser-assisted *in situ* keratomileusis (LASIK) for the correction of myopia and myopic astigmatism since 2012, and was approved by the U.S. Food and Drug Administration (FDA) in 2016^9^. In the SMILE procedure, the lenticule is cut by a femtosecond laser, and is extracted through a small arcuate incision^10^. This thin, extracted stromal lenticule may be used for other purposes. It has been described to be used as a corneal patch graft for the management of corneal micro-perforation or partial-thickness corneal defect^11^, and for the treatment of keratoconus or hyperopia, by transplanting the lenticule into stroma^12–15^. No postoperative complications, such as allogeneic rejection or corneal haze, were reported in these studies^11–15^, although the sample size was small.

The concept of tissue addition may also be applied for the correction of presbyopia. In a myopic-SMILE procedure, the extracted lenticule is convex-shaped to flatten the central cornea. By implanting the central portion of a convex-shaped lenticule, the corneal anterior curvature theoretically can be reshaped to be more hyper-prolate, hence enhancing near and intermediate vision^16^. However, even though lenticule implantation can be performed in an autogeneic manner, in reality, the majority of cases would be allogeneic, especially if they are being used for presbyopia correction. In order to increase the potential lenticule sources from autogeneic, to allogeneic, efforts have been made to reduce lenticule immunogenicity to minimize the risk of stromal immunological rejection^17^. Our group has recently published a protocol to decellularize stromal lenticules with a good preservation of the transparency, extracellular matrix content, and stromal architecture with 0.1% sodium dodecylsulfate (SDS)^17^. The use of decellularized biological inlays may offer advantages over synthetic inlays in the aspect of biocompatibility.

In the present study, we aimed to evaluate the feasibility, refractive outcomes and tissue response of using intrastromal lenticules for correction of presbyopia. We also examined the safety and feasibility in the use of decellularized xenogeneic lenticules.

## Results

### Slit lamp biomicroscopy evaluation

**Figure 1:**
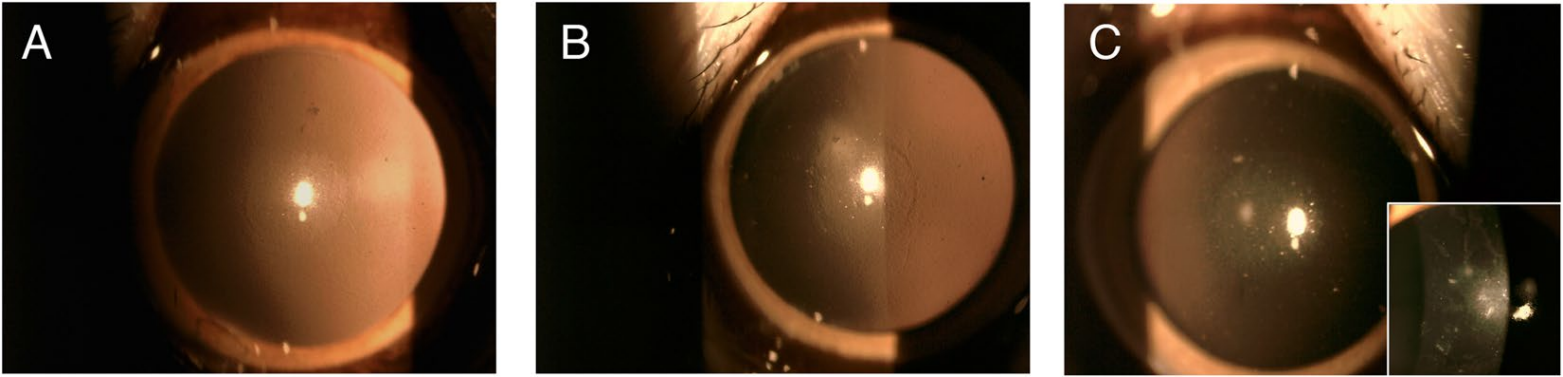
Representative slit lamp biomicroscopy pictures showing the implanted lenticule was well-centered in all eyes. The cornea remained clear in the autogeneic (**A**) and decellularized xenogeneic groups (**B**) during the study period of 6 months, while 1 eye in the xenogeneic group developed stromal rejection with stromal edema, haze and infiltrates 3.5 months after implantation (**C**, right corner), and the haze lasted for 6 months (**C**, center).

In all eyes, mild corneal edema was observed around the implanted lenticule at the first postoperative week and gradually subsided thereafter. The implanted lenticule was well-centered. The cornea remained clear throughout the study period of 6 months for the autogeneic and decellularized xenogeneic groups, whereas 1 eye in the xenogeneic group developed stromal edema, infiltrates and haze 3.5 months after implantation (Fig. 1). At 6 months, the implanted lenticules were still visible and had integrated with the surrounding stroma in all eyes.

### Anterior segment optical coherence tomography (ASOCT) evaluation, corneal thickness changes and intraocular pressure (IOP) evaluation

**Figure 2:**
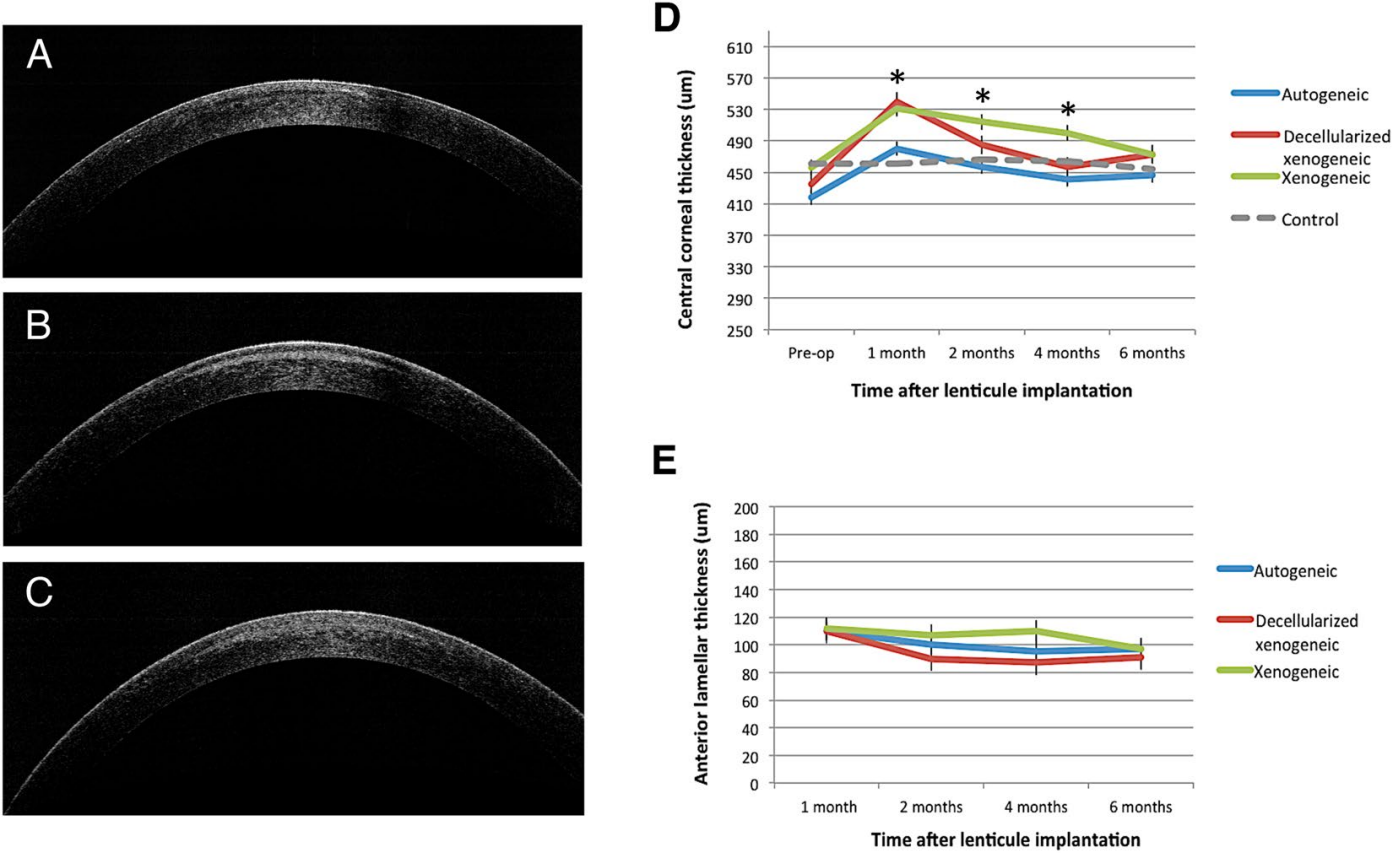
Representative ASCOT pictures at 6 months postoperatively showing the implanted lenticules. The lenticule was hypo-reflective in the autogeneic group (**A**), and more hyper-reflective in the decellularized xenogeneic (**B**) and xenogeneic groups (**C**). The line graphs showed the changes in the CCT (**D**) and anterior lamellar thickness (**E**) over the study period for different groups. * indicates *P* < 0.05 among three groups.

On ASOCT evaluation, the contour of implanted lenticule was discernible in all eyes throughout the study period. In the autogeneic group, the lenticules had less density than that of surrounding stromal tissue, while in the xenogeneic groups, either decellularized or non-decellularized xenogeneic group, the lenticules had hyper-density (Fig. 2A–C). For the eye in the xenogeneic group that developed stromal haze, there were diffuse hyperreflective spots around the lenticule.

The changes in central corneal thickness (CCT) over time are presented in Fig. 2D. The CCT increased from 418.2 ± 25.3 μm to 480.6 ± 33.3 μm, from 435.5 ± 23.8 μm to 540.9 ± 48.8 μm, and from 456.1 ± 28.6 μm to 531.6 ± 29.1 μm, at 1 month postoperatively, for the autogeneic, decellularized xenogeneic and xenogeneic groups, respectively. The CCT gradually decreased thereafter in the autogeneic and decellularized xenogeneic groups, whereas corneas with xenogeneic lenticule implantation had significantly thicker CCT than the other two groups (*P* < 0.05 at all time points except at 6 months). At 6 months, the CCT increased by 29.5 ± 3.6 μm, 38.3 ± 4.2 μm and 36.8 ± 4.3 μm for the autogeneic, decellularized xenogeneic and xenogeneic groups, respectively, due to the stromal expansion from the lenticule implantation (*P* = 0.41). The anterior lamellar thickness was 97.6 ± 5.8 μm, 91.3 ± 4.9 μm and 97.9 ± 5.1 μm for the autogeneic, decellularized xenogeneic and xenogeneic groups (*P* = 0.56), respectively (Fig. 2E).

The IOP measurement at 6 months was 10.2 ± 1.9 mmHg, 10.9 ± 1.3 mmHg and 10.4 ± 0.9 mmHg, from a preoperative value of 7.9 ± 1.1 mmHg, 8.9 ± 0.6 mmHg and 8.2 ± 1.0 mmHg, for the autogeneic, decellularized xenogeneic and xenogeneic groups, respectively. The trend of IOP increase was also seen in the control group, where the mean IOP increased from 8.2 mmHg to 10.5 mmHg. The percentage of increase was comparable across the four groups (*P* = 0.38).

### Visante Omni topographic evaluation

**Figure 3:**
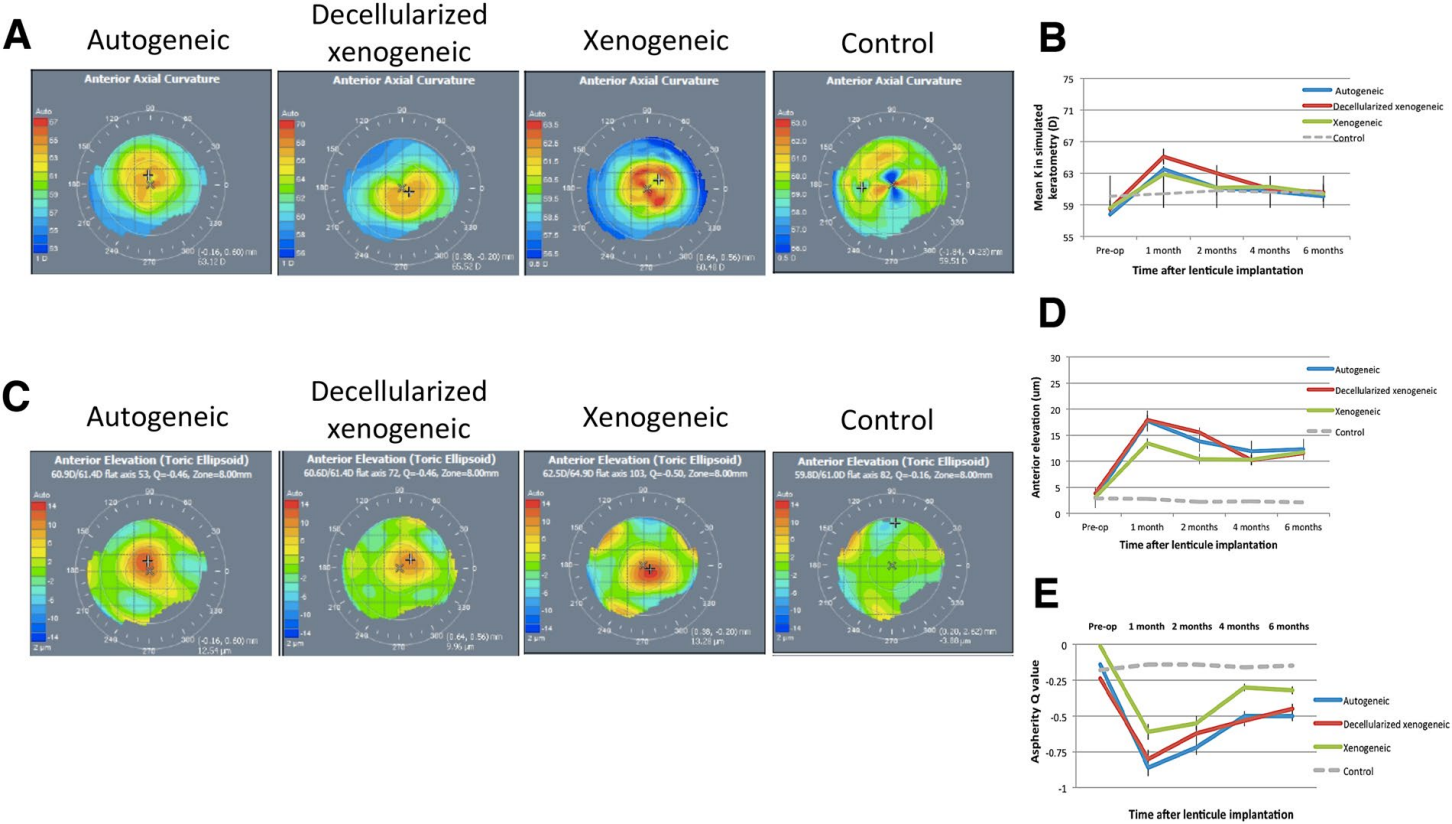
Representative anterior axial curvature maps and anterior elevation maps of ATLAS corneal topographer images at 6 months postoperatively for different groups showed central corneal steepening and central anterior elevation (**A**,**C**; note: the maximal color scale in each image was different). The line graphs showed the changes in the values of the Sim K, anterior elevation and asphericity Q over time for different groups (**B**,**D**,**E**).

The anterior axial curvature map, which describes the overall corneal power, showed a central corneal steepening in all eyes (Fig. 3A). At 6 months, the simulated keratometric (Sim K) value increased by 2.3 ± 0.4 D, 2.1 ± 0.2 D and 1.8 ± 0.3 D for the autogeneic, decellularized xenogeneic and xenogeneic groups, respectively (*P* = 0.26), whereas the Sim K value in the control group remained at the same level (Fig. 3B). The anterior elevation map showed that the mean central corneal height increased by 9.3 ± 1.1 μm, 7.7 ± 1.4 μm and 8.6 ± 0.9 μm, for the autogeneic, decellularized xenogeneic and xenogeneic groups, respectively, at 6 months after lenticule implantation (Fig. 3C,D; *P* = 0.31), with progressively less change at larger radii. The elevation returned to the level comparable to that in the untreated control eyes at 4.8 mm, 4.5 mm and 5.3 mm from the center of implanted lenticule (*P* = 0.58), indicating the effective zone of a 3 mm-implanted lenticule was 1.6, 1.5 and 1.8 times of the lenticule physical diameter, in the autogeneic, decellularized xenogeneic and xenogeneic groups, respectively. The asphericity Q value changed towards more negative after lenticule implantation in all eyes, indicating a corneal central hyper-prolate shift. The mean Q value changed by −0.36 ± 0.15, −0.26 ± 0.18 and −0.31 ± 0.22 for the autogeneic, decellularized xenogeneic and xenogeneic groups, respectively, at 6 months (*P* = 0.44; Fig. 3E). The posterior elevation maps showed that posterior corneal surface had no significant changes as compared to the preoperative levels, with the mean change at the posterior surface of −0.43 ± 0.11 μm, 0.14 ± 0.05 μm and −0.26 ± 0.08 μm at 6 months (Wilcoxon signed-rank test, all *P* > 0.05), for the autogeneic, decellularized xenogeneic and xenogeneic groups respectively. There was no significant difference in the mean change at the posterior surface across the 3 groups at 6 months (*P* = 0.38).

### *In vivo* confocal micrographs analysis

**Figure 4:**
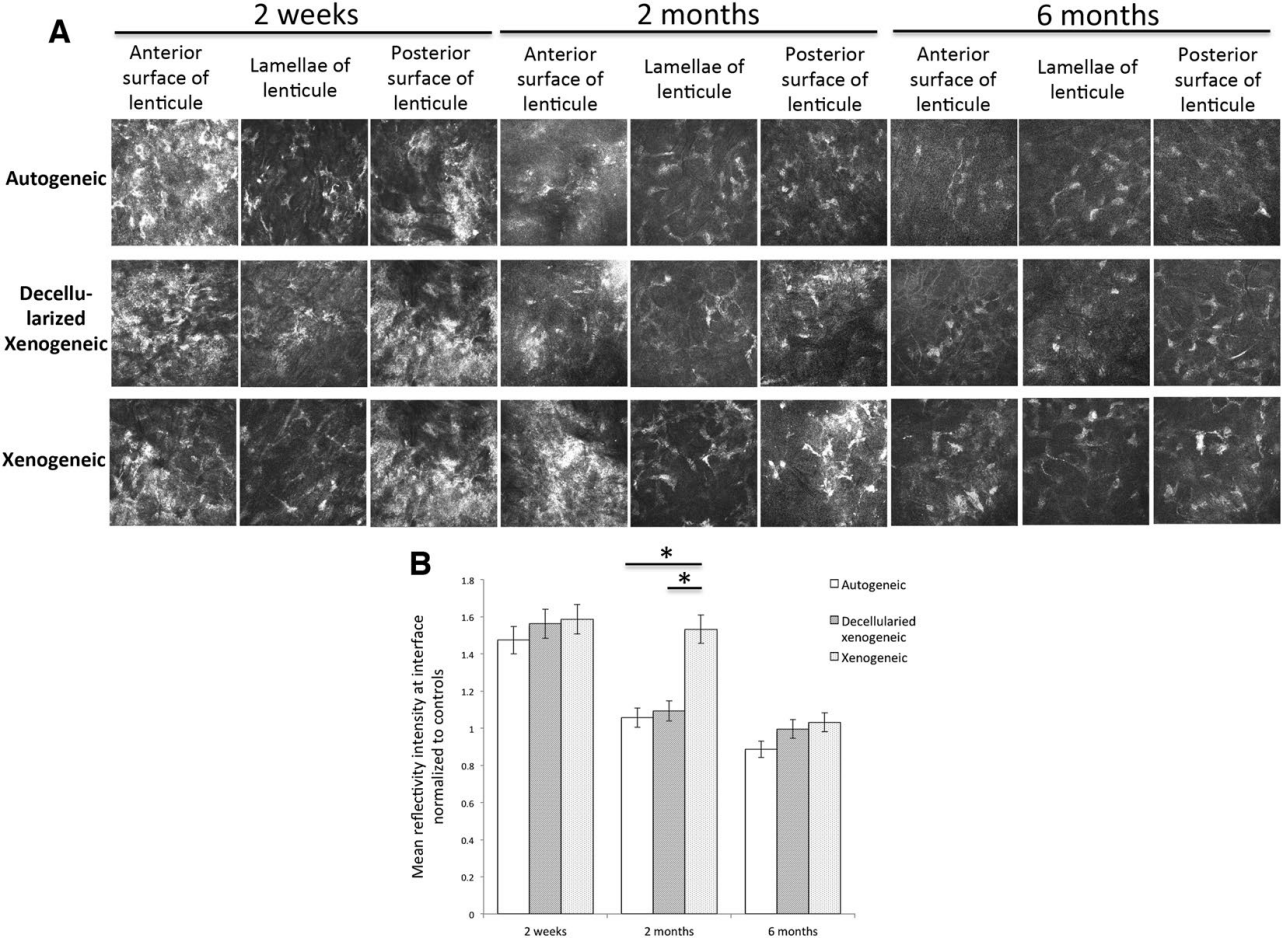
*In vivo* confocal microscopy evaluation at 2 weeks, 2 months and 6 months postoperatively at the anterior and posterior surface of lenticules, and at the lenticule plane for different groups (**A**). The bar graphs showing the mean intensity of stromal keratocytes reflectivity normalized to the controls at interface for different groups at different time points (**B**). At 2 months postoperatively, the stromal reflectivity significantly decreased to control levels in the autogeneic and decellularized xenogeneic groups, whereas the eye in the xenogeneic group that had stromal rejection had significant higher stromal reflectivity. Corneal stromal nerves were seen at both lenticule layer and stroma surrounding the lenticule at 6 months. Error bars represent SD; *indicates *P* < 0.05.

At the planes anterior and posterior to the implanted lenticule, activated keratocytes with hyper -reflectivity were seen for the initial 2 weeks in all eyes. From 1 month onwards, the keratocyte nuclei became less prominent, and the reflectivity at interfaces subsided. At 2 months postoperatively, the stromal reflectivity significantly decreased to the control levels in the autogeneic and decellularized xenogeneic groups (Wilcoxon signed-rank test; *P* = 0.045 and *P* = 0.047, respectively). One eye in the xenogeneic group had persistently high stromal reflectivity around the margin of implanted lenticule due to the development of corneal haze, but the hyper-reflectivity gradually decreased with time (Fig. 4A). At 6 months, the stromal keratocytes were quiescent in all eyes, and there was no statistically significant difference among three groups (*P* = 0.78; Fig. 4B). The keratocyte density was less in the decellularized xenogeneic group. Corneal stromal nerves were seen at lenticule layer and layers surrounding the lenticule in all groups (Fig. 4A).

### Histology and immunohistochemistry (IHC) assays

**Figure 5:**
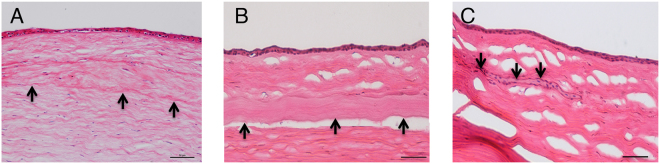
Histological section with H&E staining at 6 months showing no inflammatory cell infiltrates or fibrotic reaction around the implanted lenticules (arrows) in the autogeneic (**A**) and decellularized xenogeneic groups (**B**), while stromal cellular infiltrates (arrows) were observed around the xenogeneic lenticule (**C**). Original magnification: 100x. Scale bar: 50 μm.

**Figure 6:**
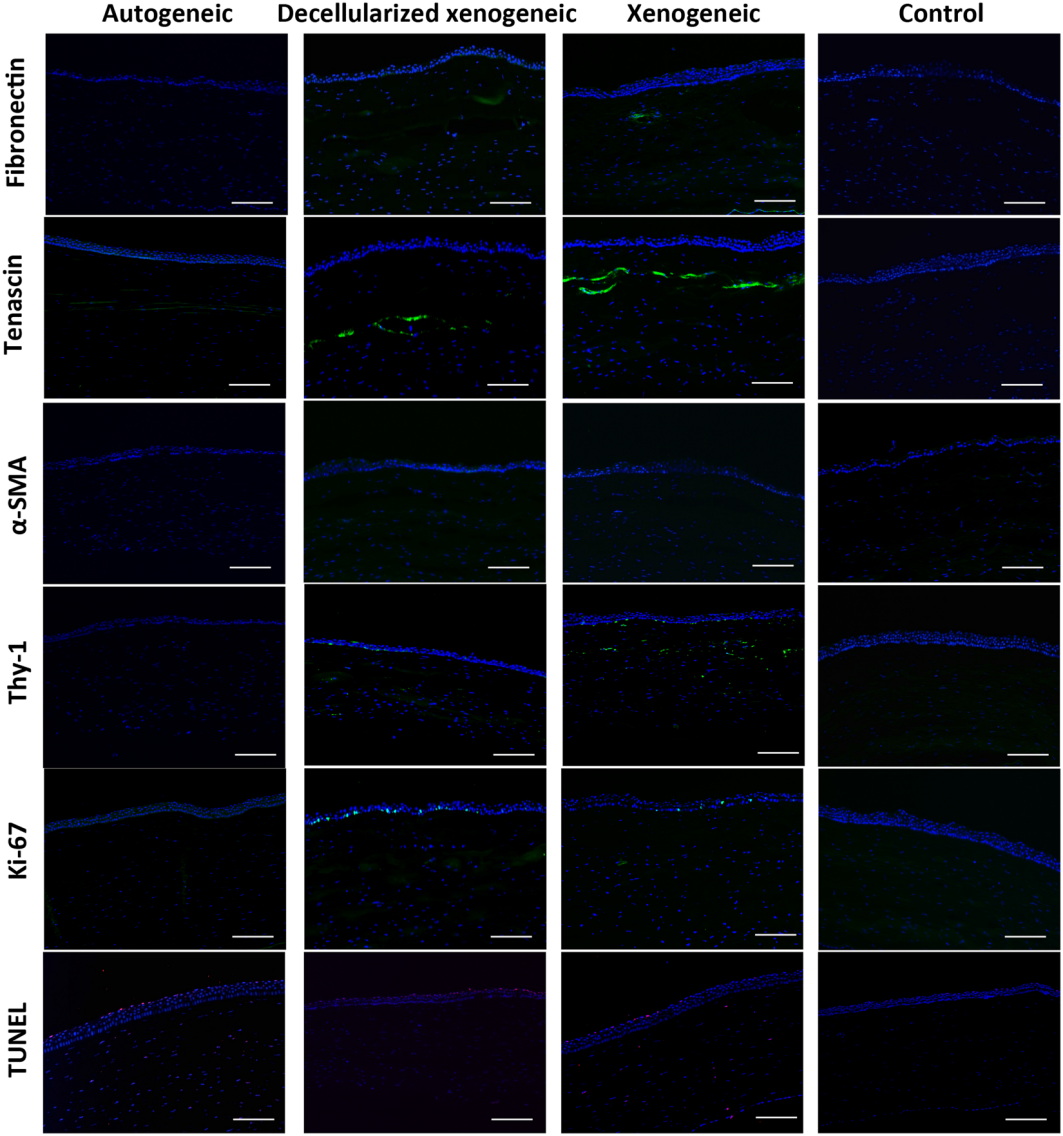
Expression of fibronectin, tenascin, α-SMA, Thy-1, Ki-67 and TUNEL assay 6 months postoperatively for different groups. There was moderate expression of tenascin in the xenogeneic groups. The staining of the rest of markers in all groups was minimal or negligible. Nuclei were counterstained with DAPI (blue). Original magnification: 100x, scale bar 50 μm.

At 6 months, hematoxylin and eosin (H&E) histochemistry revealed that there were stromal cellular infiltrates around the implanted xenogeneic lenticule, while no cellular infiltrates, fibrovascular encapsulation or stromal fibrosis, was present around the implanted lenticules in the autogeneic and decellularized xenogeneic groups, indicating good biocompatibility (Fig. 5). For IHC analysis, moderate expression of tenascin and weak expression of fibronectin were observed along the anterior surface of the implanted lenticule in the xenogeneic groups, but it was hardly seen in the autogeneic group. Scattered expression of Thy-1, a cellular marker associated with fibrosis, and scattered TUNEL positive cells, were detected within the stroma in the non-decellularized xenogeneic group. There was no α–SMA and Ki-67 staining observed in the stroma of all groups (Fig. 6).

### Transmission electron microscopy (TEM) evaluation

**Figure 7:**
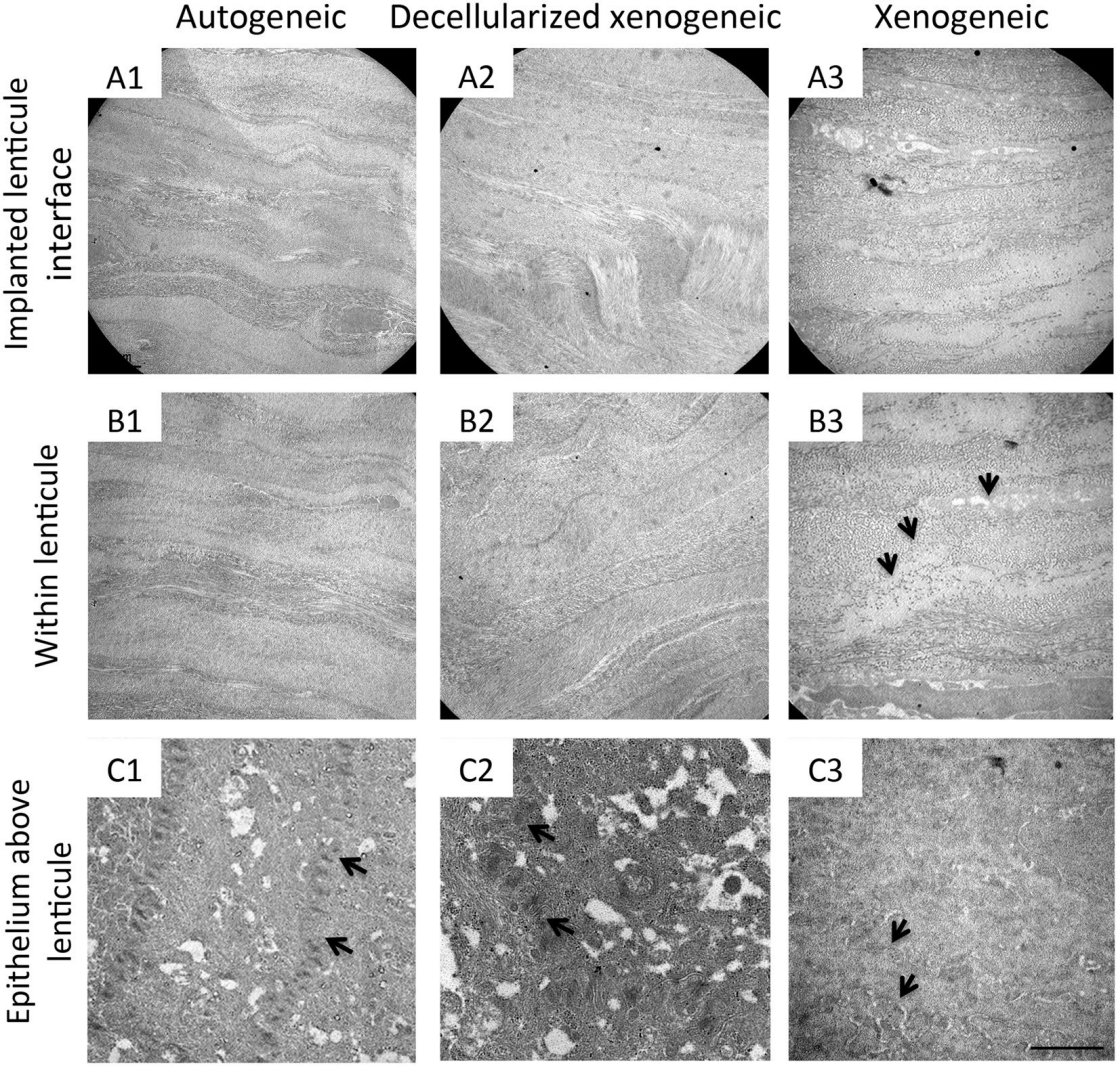
Transmission electron micrographs at 6 months showing the ultrastructural changes of the implanted lenticules in different groups. There was stromal collagen remodeling with fusiform orientation of collagen bundles at the interface in all groups (**A1**–**A3**). In the autogeneic and decellularized xenogeneic groups, the collagen fibrils within the lenticule were evenly arranged without distortion (**B1**,**B2**), but collagen fibril discontinuation and interlamellar space (arrows) were observed in the xenogeneic group (**B3**). The corneal epithelium regions above the implanted lenticules showed healthy epithelial cells with the presence of desmosomes (arrows) between cells in all groups (**C1**–**C3**). Scale bar 2 μm.

All sections from the autogeneic, decellularized xenogeneic and xenogeneic groups showed a close alignment of the implanted lenticules with the host stroma. In some regions along the interface of the implanted lenticule, the collagen fibrils appeared to have irregular, fusiform orientation and less distinct lattice arrangement, which may indicate the occurrence of stromal matrix remodeling (Fig. 7A1–3). In both autogeneic and decellularized xenogeneic groups, the extracellular matrix organization inside the implanted lenticules was similar (Fig. 7B1–2), and the collagen fibrils were evenly arranged without any distortion. In the xenogeneic group, some spaces (<0.5 μm wide) were observed along the border between the implanted and host tissues. Collagen fibril misalignment, continuation and interfibrillar and interlamellar space were also detected inside the xenogeneic lenticules (Fig. 7A3,B3). The corneal epithelium regions located directly above the implanted lenticules in all groups showed healthy epithelial cells with clear stratifications and close intercellular contacts as seen by the presence of desmosomes (Fig. 7C1–3).

## Discussion

In the present study, we demonstrated that implanting a lenticule derived from SMILE has the potential for the management of presbyopia. It effectively resulted in corneal central steepening, central hyper-prolate changes, reasonable corneal anterior surface elevation and effective zone, and acceptable predictability with no differences in the autogeneic and decellularized xenogeneic groups. The procedure had good biocompatibility and was not associated with adverse effects such as corneal haze or keratolysis. The decellularization process had the potential to reduce the occurrence of stromal rejection and did not alter the treatment efficacy.

Biological corneal inlay implantation is essentially a tissue additive procedure and has the potential advantages over corneal stromal laser ablation procedures in terms of the reversibility^4^. In this study, the implanted lenticule, derived from a SMILE procedure, acted as a shape-altering inlay to induce a corneal hyper-prolate change, by increasing the central corneal radius of curvature. Corneal lenticules extracted from SMILE offer an opportunity to utilize this ‘by-product’ for future implantation. Recently, Jacob *et al*. described their pilot study of 4 patients who underwent implantation of allogeneic corneal inlay prepared from a SMILE lenticule^16^. The authors reported that there was an improvement in the uncorrected near visual acuity in all eyes without changes in the uncorrected distance visual acuity. No regression in the near visual acuity was observed over the 6-month follow-up period although longer-term evaluation was required. Before conducting a clinical trial, we felt several further issues needed to be answered. Our study further described the thickness changes of corneal anterior lamellae, topographic changes on the anterior and posterior corneal surface, corneal wound healing and stromal keratocytes response, as well as ultrastructural changes in collagen fibrils after implantation. We also evaluated the feasibility and efficacy of the implantation of decellularized xenogeneic lenticules.

On ASOCT evaluation, the anterior central lamellae thinned by 23–29 μm, and the resultant total corneal thickness increased by 29–36 μm after implanting a 65 μm lenticule. This is similar to previous studies using the Raindrop shape-changing corneal inlay, where authors showed a 18 μm thinning in the central epithelial thickness after the Raindrop inlay implantation^18^. The thinning resulted from the postoperative epithelial remodeling, a common wound healing response after a refractive procedure^19^. The magnitude of the epithelial thinning was proportional to the curvature of the anterior corneal surface height change^19–21^. The anterior elevation maps in our study showed that the changes in the anterior corneal surface height gradually decreased from the center to periphery, and the effective zone determined by the anterior elevation map was 1.5–1.8 times of the lenticule physical diameter. The fact that the effective zone extends beyond the lenticule diameter mainly comes from the effect of epithelial wound remodeling^18^. Further analysis on corneal epithelial profiles would be worth including in future clinical studies.

The refractive predictability following lenticule implantation, either for hyperopia or presbyopia treatment, warrants investigation. The ultimate alteration of corneal refractive power depends on the thickness of implanted lenticule as well as the depth of implantation, and the postoperative corneal wound healing response. From a large-scaled clinical study on the Raindrop inlay, the depth of implantation was suggested to be at between 31 to 34% of the central corneal thickness because it yielded the least rate of corneal haze but still provided sufficient effect on the change of anterior corneal shape^6^. Hence the anterior cap thickness of the pocket in the present study was set at 120 μm. In a monkey study in which the authors implanted a −4.0 D lenticule under a 100 μm flap, the final corneal refractive power changes was 0.7 D less than the predicted 4.0 D correction^22^. Similar finding was seen in a case report using a −10.5 D lenticule to correct a spherical equivalent refraction of +10.25 D hyperopia. The final refractive reduction was only 50% of the intended correction due to the corneal posterior surface changes and epithelial remodeling^23^. In the present study, the implanted lenticule did not change the posterior corneal surface, as the lenticule was thin and implanted at the anterior one-fourth depth. The final keratometric reading of the central 3 mm cornea increased by 1.8 to 2.3 D after implanting the central 3 mm of a −3.0 D lenticule. This was reasonable as it falls in the range of loss of accommodation in patients. This was also consistent with the data showing that the keratometric reading of the central 3 mm cornea changed by 2.2 D at 3 months following Raindrop inlay implantation^24^ although the central thickness of the Raindrop inlay is thinner than that of the inlays we used in the present study (32 μm versus 65 μm)^4^. It might be due to different stromal wound remodeling responses between synthetic and biological inlays as biological inlays are assumed to have more tissue integration. It might also indicate that, as compared to synthetic inlays, the thickness of biological inlays to be implanted may be required to be thicker to achieve the same long-term treatment efficacy as synthetic inlays. This warrants further comparative studies to confirm. In future clinical trials, how the added power from biological inlays helps on near vision, as well as whether the inlays affect distance vision, will be studied. From the FDA clinical trial on the synthetic Raindrop inlays, the monocular uncorrected distance visual acuity (UDVA) decreased by 1.2 lines after implantation, but the mean binocular UDVA was comparable to the preoperative levels^6^. Decrease in the inlay diameter may attenuate the impact on the UDVA, but it may also compromise the effects on the added power for near vision.

Lenticule implantation is essentially a selective lamellar keratoplasty procedure, hence theoretically it still carries the risk of rejection if the lenticule is not autogeneic. However, the risk of rejection is expected to be low, compared to full-thickness or lamellar corneal transplantation, because (1) the lenticule was only 65 μm at the thickest point and 3 mm in size, and therefore the antigenic load would be small, to elicit an immunological response (2) the lenticule was composed of stromal keratocytes/collagen and no epithelial or endothelial cells, which are more antigenic^25^ (3) the lenticule was protected in the stromal bed and was not in contact with tears, limbus, or aqueous humor that contain stimulating factors to trigger immune rejection^25^. To minimize the occurrence of stromal rejection, our group has optimized the decellularization protocol using human lenticules to effectively reduce stromal immunogenicity^17^. Due to the strict regulation on the number of non-human primates to be used, we decided to apply the decellularization process to xenogeneic lenticules, rather than allogeneic lenticules. It is well-known that the elicited rejection response is stronger in xenogeneic than allogeneic implantation^26^, hence it was expected that the stromal immunological reaction in allogeneic implantation could also be suppressed by decellularization if the decellularization process has effects on the xenogeneic groups. For the synthetic Raindrop inlay, it was reported that the postoperative corneal haze mainly occurred in the first 6 months after implantation, hence the study time point set in the present study was 6 months. One eye in the xenogeneic control group (i.e. non-decellularized group) developed stromal rejection 3.5 months after implantation. This also accounted for the significantly thicker corneal thickness in the xenogeneic group compared to the other two groups. In order to understand the nature course of the immunological response that non-decellularized xenogeneic lenticules induced and to more accurately compare the immune reaction among three groups, the postoperative steroid regime was standardized and no additional steroid was given in the non-decellularized xenogeneic group even though the stromal rejection occurred. We also noticed that the decellularization process, prior to implantation, resulted in lenticule tissue edema (Fig. 2D), but this resolved with time.

The confocal microscopy, IHC, and TEM results confirmed the implanted biological lenticules were biocompatible and had good integration into the host stroma, which are the two concerns when using synthetic corneal inlays^27^. The stromal keratocyte response evaluated by *in vivo* confocal microscopy subsided to normal levels after 1 month postoperatively in the autogeneic and decellularized xenogeneic groups. These two groups also only elicited minimal to moderate expression of tenascin, a marker for corneal inflammation and fibroblastic fibrosis activity^28^, whereas the xenogeneic lenticules presented distinct expression of tenascin.

The development of intrastromal inlays has long posed a challenge with respect to nutrient passage across the inlay. Early synthetic impermeable inlays resulted in anterior stromal thinning or keratolysis because of insufficient fluid flow and nutrition transport from the aqueous^7^. Biological inlays, proposed in the present study, theoretically offer superior permeability and biocompatibility than synthetic inlays. Under TEM, the corneal epithelium maintained normal structures including the tight junction network in all eyes, suggesting no degeneration of epithelial layer due to impedance of nutrient or metabolic gradient. The collagen misalignment and discontinuation inside the xenogeneic lenticules might be the consequence of stromal edema due to stromal rejection. Lastly, the limitation of the present study was the small sample size because of the strict ethical regulation on the use of non-human primates. However, it was scientifically and ethically important to conduct pre-clinical studies before clinical trials, and a non-human primate is the most appropriate animal model for the present study as primates share the highest ocular similarities, such as corneal thickness^29^ and the presence of a thick Bowman’s membrane (as opposed to a rabbit model)^30^, as well as genetic homologies, with human.

In conclusion, our study showed the safety and efficacy of the use of SMILE lenticules for the management of presbyopia. The decellularization process may increase the potential utilization of lenticules without changing the efficacy. The results provide information for the design of future clinical trials.

## Methods

### Study animals and experimental groups

Ten 2- to 5-year-old Macaca fascicularis (cynomolgus macaque) non-human primates (n = 20 eyes) were randomly allocated to four groups: autogeneic (n = 6 eyes), decellularized xenogeneic (n = 6 eyes), xenogeneic (n = 6 eyes), and control groups (no procedure performed; n = 2 eyes). All animals were treated according to the guidelines of the Association for Research in Vision and Ophthalmology (ARVO) Statement for the Use of Animals in Ophthalmic and Vision Research. The protocol was approved by the Institutional Animal Care and Use Committee (IACUC) of SingHealth, Singapore. The study design of using bilateral surgery in the protocol was approved by IACUC, as the surgery was not visually disabling procedure, and adhered to ARVO statement for the use of animals in research. During surgeries and evaluations, the monkeys were tranquilized intramuscularly with ketamine hydrochloride (10 mg/kg) or medetomidine (0.02 mg/kg). Anaesthesia was induced with 2–3% inhaled isoflurane and maintained with 1–2% inhaled isoflurane. All surgical procedures were performed by an experienced refractive surgeon (J.S.M.).

### SMILE and lenticule implantation procedures

For the autogeneic group, one eye of each animal was randomly selected for the SMILE procedure. SMILE was performed using a previously described technique^31^. In brief, a myopic SMILE correction of −3.0 D was performed using a 500-kHz femtosecond laser (Visumax; Carl Zeiss Meditec, Jena, Germany). The eye was docked on a small curved interface suction cone. The laser parameters were: 120 μm cap thickness, 7.5 mm cap diameter, and 6.5 mm lenticule diameter, with the laser energy at 170 nJ. The lenticule was grasped and removed by a Tan DSAEK forceps (ASICO, Westmont, IL, USA), and was then spread out and dried with a surgical sponge. A 3 mm trephine (World Precision, Sarasota, FL, USA) was subsequently centered, in the middle of the lenticule, to fashion a corneal inlay. The inlay was 65 μm in thickness in the center and then implanted into the contralateral eye, in a 7.5 mm intrastromal pocket created by the Visumax femtosecond laser, at the depth of 120 μm and over the pupillary center.

For the decellularized xenogeneic and xenogeneic groups, freshly enucleated (<6 hours from death) porcine eyes obtained from a local abattoir (Primary Industries Pte Ltd, Singapore) were used. The porcine −3.0 D lenticules were obtained as described above. Half (n = 3) of these xenogeneic lenticules were treated with 0.1% SDS (Sigma-Aldrich, St. Louis, MO, USA) for 24 hours under agitation (300 r.p.m), followed by washes with phosphate-buffered saline (PBS) 3 times, each again for 24 hours under agitation, whereas the other half (n = 3) of xenogeneic lenticules had no SDS treatment. All eyes received topical Tobradex ointment (Alcon, Fort Worth, TX, USA) two times daily for 1 week after surgery.

### Clinical evaluation

All eyes underwent clinical evaluation at day 4, week 1, week 2, month 1 and monthly thereafter until month 6 postoperatively, with slit lamp biomicroscopy (Nikon FS-3V; Nikon, Tokyo, Japan), tonopen (Tono-Pen AVIA, Reichert, NY, USA) for IOP measurement, ASOCT (RTVue; Optovue, Inc, Fremont, CA), Visante Omni (Carl Zeiss Meditec, Jena, Germany), and *in vivo* confocal microscopy (IVCM; HRT3; Heidelberg Engineering GmbH, Heidelberg, Germany). Five IOP measurements were taken for each eye. For ASOCT evaluation, three high-resolution corneal cross-sectional scans (8 mm scan length, single scan mode) were obtained for each eye at each time point, and the CCT as well as central anterior lamellar thickness was measured by an independent observer (H.P.A). The IVCM and Visante Omni scans were performed as described previously^32,33^. The IVCM micrographs were also further analyzed by selecting 3 micrographs from the planes anterior and posterior to the implantation lenticule each and semi-quantifying the mean gray value of reflectivity using Image J (http://imagej.nih.gov/ij/; provided in the public domain by the National Institutes of Health, Bethesda, MD, USA)^34^. The micrographs of the lenticule plane were not included for the reflectivity analysis as the density of stromal keratocytes was one of the determinants of the gray value of reflectivity, and the density was expected to be less in the decellularized group than the other two groups^17^.

### Histology and immunohistochemistry (IHC)

At 6 months postoperatively, the monkeys were euthanized under anesthesia, and the corneas were excised. The corneas were embedded in an optimal cutting temperature compound at −80 °C and cryosectioned at 5 μm thickness. The sections were then processed for H&E histochemistry and visualized under light microscopy (Axioplan 2, Carl Zeiss, Oberkochen, Germany). For IHC, after blocking with bovine serum albumin (2%, Sigma-Aldrich, St. Louis, MO, USA) and saponin-permeabilization, the sections were incubated with primary antibodies against cellular fibronectin (2 μg/ml; Millipore, Billerica, MA, USA), tenascin-C (1 μg/ml; Abcam, Cambridge, UK), smooth muscle actin (α-SMA; 2 μg/ml; Dako Cytomation, Glostrup, Denmark), Thy-1 (1 μg/ml; BD Biosciences, CA, USA), and Ki67 (1 μg/ml; Dako Cytomation, Glostrup, Denmark) in PBS with 1% bovine serum albumin, 0.15% saponin, 0.0001% Triton X-100 and Tween 20 for 2 hours at room temperature. After washing with PBS, they were labelled with Red-X or Alexa488-conjugated immunoglobulin G secondary antibody (Jackson ImmunoRes Lab, West Grove, PA, USA). Samples were mounted with UltraCruz mounting medium containing DAPI (Santa Cruz Biotechnology, Dallas, TA, USA) and viewed under fluorescence microscopy (AxioImager Z1, Carl Zeiss, Oberkochen, Germany). To detect apoptosis, a fluorescence-based terminal deoxynucleotidyl transferase dUTP nick end labeling (TUNEL) assay (*In Situ* Cell Death Detection Kit, Roche, Basal, Switzerland) was used according to the manufacturer’s instructions. Quantification of TUNEL positive cells was performed on 5 randomly selected regions for each sample at 100x magnification by a single masked observer (E.T-W.P.).

### Transmission electron microscopy

A half of excised corneas were fixed in 3% glutaraldehyde (Electron Microscopy Sciences, Hatfield, PA, USA), 1% tannic acid (Sigma-Aldrich, St. Louis, MO, USA) and 1% aqueous solution of osmium tetroxide, and then were processed for Epon Aradite embedding and ultrathin sectioning at 90 nm thickness. After staining with 3% uranyl acetate and lead citrate, the sections were examined under TEM (JEOL 2100, Tokyo, Japan).

### Statistical analysis

All data were expressed as mean ± standard deviation (SD). Statistical comparisons among three groups were performed using Kruskal–Wallis test with Dunn post-hoc tests. A Wilcoxon signed-rank test was used for the comparison between values before and after implantation. Statistical analyses were performed using STATA software (version 13, STATACrop, College Station, TX). *P* values less than 0.05 were considered statistically significant.
